# Activity of peroxisomal enzymes, and levels of polyamines in LPA-transgenic mice on two different diets

**DOI:** 10.1186/1476-511X-4-23

**Published:** 2005-10-04

**Authors:** Knut A Eliassen, Bjørn P Brodal, Aud Svindland, Harald Osmundsen, Helle Rønning, Srdjan Djurovic, Kåre Berg

**Affiliations:** 1 Department Basic Sciences and Aquatic Medicine, Norwegian School of Veterinary Science, P.O. Box 8146 Dep. No-0033 Oslo, Norway; 2 Department of Oral Biology, University of Oslo, P.O. Box 1052 Blindern, No-0316 Oslo, Norway; 3 Department of Pathology, Aker University Hospital, No-0514 Oslo, Norway; 4 Institute of Medical Genetics, University of Oslo, P.O. Box 1036 Blindern, Oslo, Norway; 5 Department of Medical Genetics, Ullevål University Hospital, No-0407 Oslo, Norway

## Abstract

**Background:**

In man, elevated levels of plasma lipoprotein (a)(Lp(a)) is a cardiovascular risk factor, and oxidized phospholipids are believed to play a role as modulators of inflammatory processes such as atherosclerosis. Polyamines are potent antioxidants and anti-inflammatory agents. It was therefore of interest to examine polyamines and their metabolism in LPA transgenic mice.

Concentration of the polyamines putrescine, spermidine and spermine as well as the activity of peroxisomal polyamine oxidase and two other peroxisomal enzymes, acyl-CoA oxidase and catalase were measured. The mice were fed either a standard diet or a diet high in fat and cholesterol (HFHC). Some of the mice in each feeding group were in addition given aminoguanidine (AG), a specific inhibitor of diamine oxidase, which catalyses degradation of putrescine, and also inhibits non-enzymatic glycosylation of protein which is implicated in the aetiology of atherosclerosis in diabetic patients. Non-transgenic mice were used as controls.

**Results:**

Intestinal peroxisomal polyamine oxidase activity was significantly higher in LPA transgenic mice than in the non-transgenic mice, while intestinal peroxisomal catalase activity was significantly lower. Hepatic β-oxidation increased in Lp(a) transgenic mice fed the HFHC diet, but not in those on standard diet.

Hepatic spermidine concentration was increased in all mice fed the HFHC diet compared to those fed a standard diet, while spermine concentration was decreased. With exception of the group fed only standard diet, transgenic mice showed a lower degree of hepatic steatosis than non-transgenic mice. AG had no significant effect on hepatic steatosis.

**Conclusion:**

The present results indicate a connection between peroxisomal enzyme activity and the presence of the human LPA gene in the murine genome. The effect may be a result of changes in oxidative processes in lipid metabolism rather than resulting from a direct effect of the LPA construct on the peroximal gene expression.

## Background

Elevated levels of plasma lipoprotein (a) Lp(a) are a significant cardiovascular risk factor in man [1]. We have earlier reported development of arteriosclerosis in aorta of mice transgenic for cDNA representing the human gene for Lp(a), hLPA, on a standard diet [2,3], while the non-transgenic mice only sporadically developed arteriosclerosis. In the LPA cDNA-transgenic animals, apolipoprotein(a), (apo(a)), occurs free in plasma, and we found a significant correlation between the plasma apo(a) concentration and the size of aortic lesions.

The present investigation, based on tissue samples from the animals in the above-mentioned study, was undertaken to uncover if polyamines could influence on the atherosclerotic development. There were no leftovers of blood and the aortic wall for polyamine measurements. The liver and kidney were therefore chosen.

The rationale for examining the polyamines, spermidine and spermine, is that their positive charges strongly interact with phospholipids and inhibit lipid peroxidation [4], and to a certain extent protect liposome from oxidation [5]. In addition, spermine exhibits an anti-inflammatory effect [5] and exerts an antagonistic action on platelet aggregation [6]. Since cell proliferation in the vascular walls is important in the development of atherosclerotic lesions, knowledge of levels and metabolism of polyamines are *per se *of interest because of their well-known importance for cell growth and differentiation. In addition it was of interest to measure the activity of the peroxisomal polyamine oxidase, an enzyme important in converting polyamines [7]. Examination of the activity of peroxisomal enzymes was of interest also because the peroxisomal proliferators activated receptors (PPARs) are known to be involved in the development of arteriosclerosis (for review, see [8]. We have earlier shown that feeding rats a diet enriched in polyamines resulted in a decrease in hepatic polyamine oxidase and catalase activity, which could be restored by simultaneously supplementing the diet with clofibrate, a peroxisomal proliferator and a hypolipidemic drug [9].

The fact that the hypolipidemic drug, clofibrate, changed the activity of polyamine oxidase indicates that the fat content of tissues may influence polyamine metabolism. It was therefore of special interest to examine if fat loading had any effect on liver polyamine content, and on polyamine oxidase activity.

In the present study we found that polyamine oxidase activity was higher in transgenic mice than in non-transgenic animals. In order to examine whether this reflects a general increase in peroxisomal enzymes, we measured the activity of two other peroxisomal enzymes, catalase and β-oxidase. Catalase decomposes H_2_O_2, _a product of oxidase activity, and thereby protects the cell against the toxic effect of H_2_O_2, _while peroxisomal β-oxidation plays the important physiological role of oxidation very long fatty acids and the side chain of cholesterol.

Some groups of mice were treated with AG because AG inhibits the formation of non-enzymatic glycosylation of proteins [10], which are implicated in the aetiology of diabetic complications, including arteriosclerosis [10,11], and because AG is a well-known specific inhibitor of diamine oxidase, which catalyses degradation of putrescine, the precursor of the polyamines; spermidine and spermine.

We here report that the introduction of the human LPA gene into FVB mouse resulted in no significant changes in the polyamine concentration in liver and kidneys, but it changed the activity of three peroxisomale enzymes namely: intestinal polyaminoxidase and catalase and hepatic acyl-CoA oxidase (responsible for β-oxidation). There seemed to be less hepatic steatosis in the transgenic mice compared to the control FVB mice.

In mice fed a HFHC diet compared to those fed a standard low fat diet, the hepatic spermidine and spermine concentrations were different, resulting in a lower ratio between spermidine and spermine concentrations in the HFHC fed mice.

## Results

The results did not suggest a sex difference with respect to any of the measured parameters.

### Body weight

Body-weight decreased by 2–5% during the experimental period. Animals treated with aminoguanidine showed the highest weight-loss. The reduction in the body weight may be related to the relatively high age of the mice. Mice on the high fat diet, showed an intermittent increase in body weight of 2–9%, with a peak about 3 weeks after start. However, mean body weight at the end of the treatment period, was not significantly different from those of the groups fed a standard diet (data not shown).

### Polyamine oxidase activity

**Figure 1:**
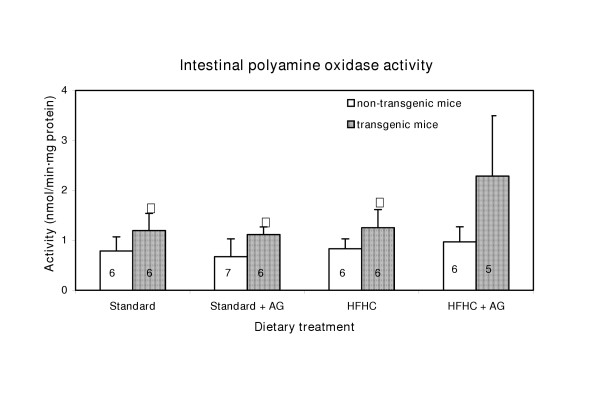
Intestinal polyamine oxidase activity in LPA transgenic and non-transgenic mice. The mice were fed two different diets, one high in fat and cholesterol (HFHC diet), the other a standard diet low in fat. Some of the animals from each group (see Table 1) were treated with 0.5% AG (aminiguanidine) in the drinking water. After 100 days of treatment the activity of the enzyme was assayed. The number of mice in each group is given with numbers within each column. Experimental details are given in Materials and Methods. The histograms represent means with SD indicated. Population means that were significantly different from the non-transgenic mice are denoted with asterisk, * (p < 0.05).

The small intestinal polyaminoxidase activity was significantly (p < 0.05) higher in the transgenic mouse groups than in the non-transgenic. The polyamine oxidase activity in the small intestine was about twice as high as in the liver. Data showing the effects of the treatments on intestinal polyamine oxidase activity are presented in Fig 1. Hepatic polyamine oxidase activity was 0.41 ± 0.06 nmol/min·mg and was not affected by the diet fat content, AG treatment, or transgenity.

### Catalase activity

**Figure 2:**
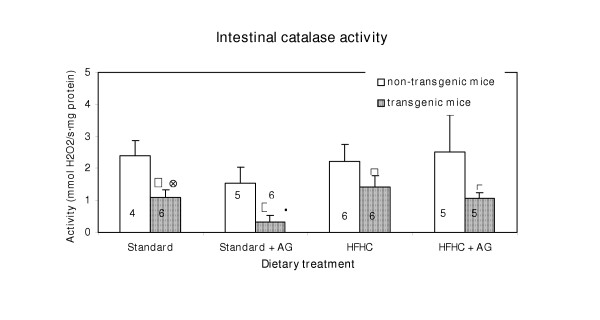
Intestinal catalase activity in LPA transgenic and non-transgenic mice. The histograms represent means with SD indicated. Population means that were significantly different from the non-transgenic mice (p < 0.05) are denoted with *. Population means denoted with • indicate difference from the corresponding mice fed the HFHC diet and ⊗ indicate significant differences from the corresponding aminoguanidine (AG) treated mice. Experimental details are given in the legend to Fig. 1 and in Materials and Methods section.

Figure 2 shows the catalase activity measured in homogenates from the small intestine. In all treatment groups the catalase activity in the transgenic animals was significant lower than in the non-transgenic mice. In transgenic mice, given aminoguanidine in the drinking water, the catalase activity was significantly higher in those on the HFHC diet than in those on the standard diet (Fig. 2). AG treatment of transgenic mice on the standard diet reduced the intestinal catalase activity significantly.

There were only minor and insignificant differences in hepatic catalase activity between transgenic and non-transgenic mice and between the two diets. The activity was roughly the same as in the small intestine of non-transgenic mice.

AG in the drinking water to mice on the HFHC diet resulted in an increased hepatic catalase activity from about 1.5 to 3.0 μmol H_2_O_2_/s·mg protein.

### Peroxisomal β-oxidation (hepatic acyl-CoA oxidase activity)

**Figure 3:**
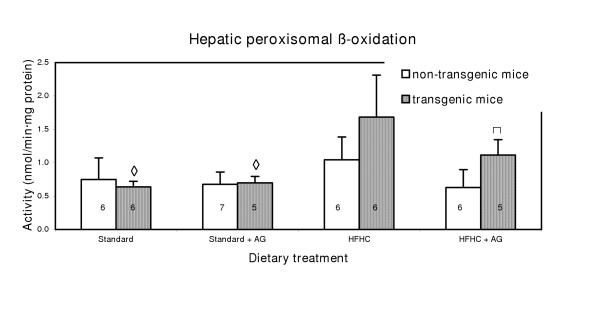
Hepatic peroxisomal β-oxidation activity in LPA transgenic mice and non-transgenic mice. Experimental details are given in the legend to Fig. 1 and in Materials and Methods section. The histograms represent means with SD indicated. Population means that were significantly different (p < 0.05) from non-transgenic mice are denoted with *. Population means denoted with ♦ indicate significant differentce from the corresponding mice fed a HFHC diet.

Peroxisomal β-oxidation in livers of transgenic mice fed the HFHC diet and aminoguanidine in the drinking water was significantly (p < 0.05) higher than in the non-transgenic mice on the same treatment (Fig. 3). In corresponding experiment with mice on the same diet, but without aminoguanidine (Fig. 3), the difference observed did not reach statistical significance.

### Intestinal diamine oxidase activity

The diamine oxidase activity in the proximal part of the small intestine was powerfully inhibited in animals given AG, irrespective of their genetic status. The mean ± SD values of diamine oxidase activity expressed as amount putrescine turnover pr. min and g tissue was 220 ± 160 and 17 200 ± 6200 pmol for the aminoguanidine treated and the non-treated animals, respectively. This is in agreement with the established inhibitory effect of aminoguanidine on diamine oxidase activity [12].

### Tissue polyamines

The hepatic concentrations of putrescine wearied around the level of detection of the assay. Evaluation of these data could therefore not be carried out.

**Figure 4:**
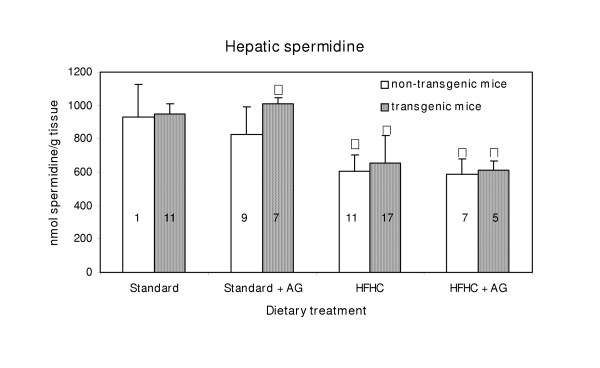
Hepatic spermidine concentration in LPA transgenic and non-transgenic mice. The columns represent means with SD indicated. Population mean that is significantly different from that of non-transgenic mice is denoted with asterisk, *. Δ Indicate significant difference from the corresponding groups fed a standard diet. Experimental details are given in the legend to Fig 1 and in the Materials and Methods section.

**Figure 5:**
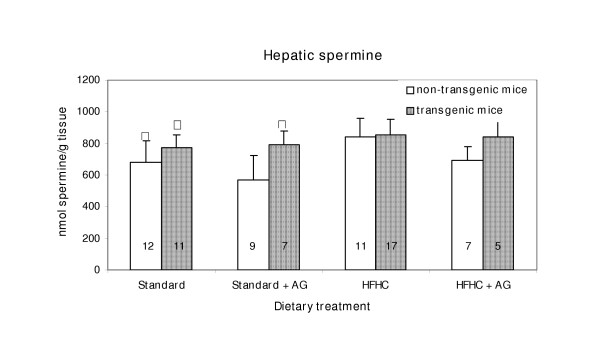
Hepatic spermine concentration in LPA transgenic and non-transgenic mice. The columns represent means with SD indicated. Population mean that is significantly different (p < 0.05) from that of the non-transgenic mice is denoted with asterisk, *. Δ Indicate significant difference (p < 0.05) from the corresponding HFHC fed groups. Experimental details are given in the legend to Fig. 1 and in the Materials and Methods section.

With exception of mice on standard diet and aminoguanidine treatment, transgenic and non-transgenic mice exhibited no significant differences in hepatic spermidine and spermine concentrations (Figs. 4 and 5)

The hepatic concentration of spermidine in mice fed the atherogenic diet, HFHC; was significantly (p < 0.05) lower than in mice given standard diet (Fig. 4). On the other hand, a slight, but significant increase (p < 0.05) in spermine concentration was observed in mice fed the HFHC diet without AG in the drinking water, compared to those fed the standard diet (Fig. 5). The spermidine/spermine ratio was 0.76 for mice on the HFHC diet, and 1.32 for those on standard diet.

In kidney there were no differences between transgenic and non-transgenic mice. Thus for the non-transgenic mice the putrescine, spermidine and spermine concentrations were (73 ± 36) nmol/g, (363 ± 83) nmol/g and (606 ± 101) nmol/g, respectively, and the corresponding values for the transgenic animals were 66 ± 22, 398 ± 170 and 642 ± 154 nmol/g. Furthermore, there was no difference between mice on the two diets, or between AG treated and untreated mice.

### Hepatic steatosis

**Figure 6:**
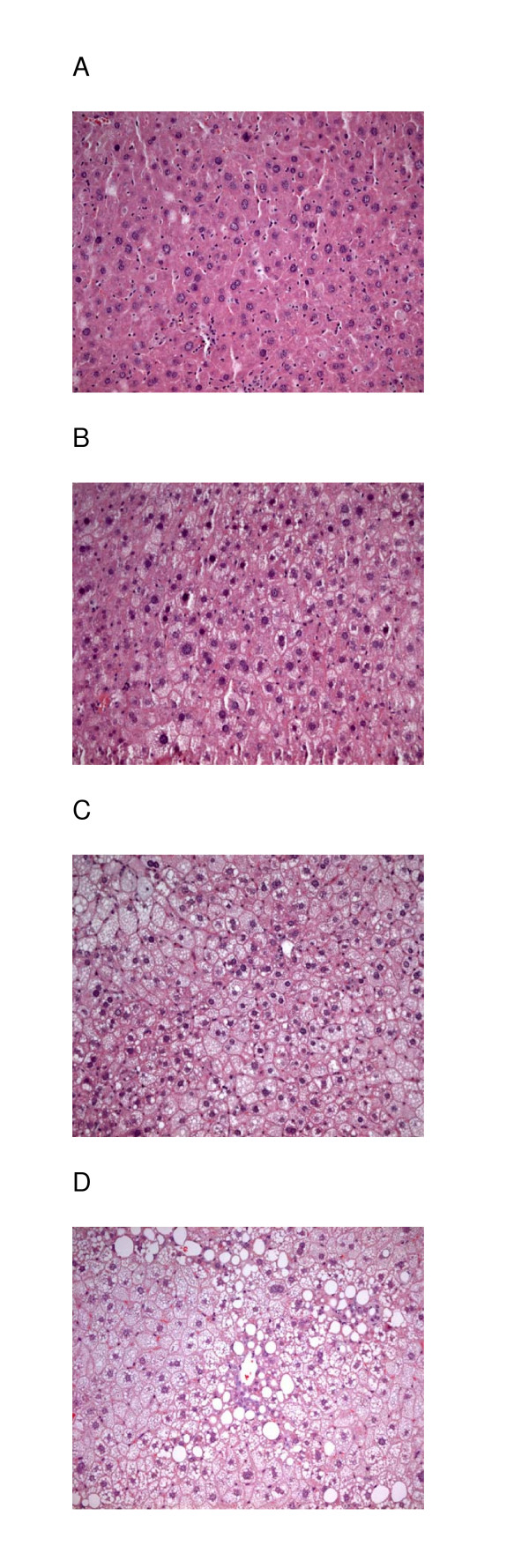
Scoring of hepatic steatosis. A) Normal liver with no lipid deposits, grade 0, B) Liver tissue with steatosis grade 1 (slight steatosis), C) grade 2, that between slight and serve stearosis, D) Liver tissue with steatosis grade 3 (serve steatisis), almost all cells are affected and many with large fat vacuoles. Magnification 1000x.

**Figure 7:**
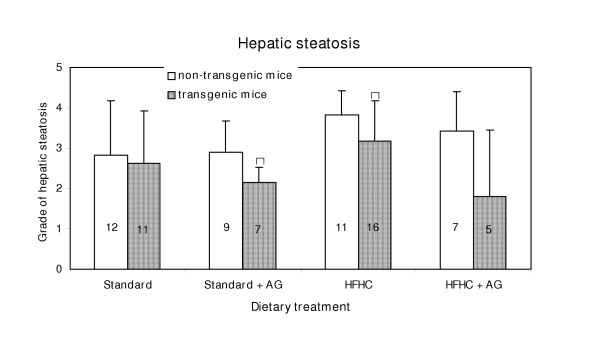
Hepatic steatosis (grade 0 to 3) in LPA transgenic and non-transgenic mice. The columns represent means with SD indicated. Population means that are significantly (p < 0.05) different from the non-transgenic mice are denoted with asterisks, *. Experimental details are given in the legend to Fig. 1 and in the Materials and Methods section.

In the fatty infiltrations, there was no sign of inflammatory cells. Hepatic steatosis was graded from 1 to 3. Typical sections of liver with steatosis of grade 1, 2 and 3, together with sections of a normal liver are shown in Fig. 6. Steatosis occurred in the liver of all, but four mice. The grade of steatosis was lower in the transgenic mice than in non-transgenic mice, but the difference was statistical significant (p < 0.05) only for those on a standard diet with AG in the drinking water and for those fed only a high fat diet (Fig. 7).

## Discussion

### Enzyme activities

Introduction of cDNA representing the LPA gene together with the transferrin promotor, into the mouse genome resulted in changes in activity of the peroxisomale enzymes polyamine oxidase and catalase in the small intestine, but not in the liver (Figs. 2, 3, 4). This is in contrast to the fact that the LPA gene is expressed in liver, but not in the intestines [13], indicating that the effect on the intestinal enzymes must be secondary. Since the genes coding for polyamine oxidase (Locus ID no Mm 212503) and for acyl-CoA oxidase (Locus ID no. Mm 11430) are located on different mouse chromosomes, 7 and 11, respectively, it is unlikely that the observed changes in these enzyme activities were caused by the introduction of the LPA gene with a transferrin promotor, in or near the regulator region of the gene for these enzymes.

The increased intestinal polyamine oxidase activity in LPA transgenic mice compared to non-transgenic mice (Fig. 1), indicates an altered peroxisomal polyamine metabolism. When the activity of the H_2_O_2 _producing polyamine oxidase enzyme is increased in the transgenic mice, an increase in the catalase activity should be anticipated. However, the opposite was the case.(Fig. 2) Increased polyamine oxidase activity can therefore not be explained by a general increase in activity of peroxisomal enzymes. The increase in H_2_O_2 _production from enhanced polyamine oxidase activity is probably marginal, compared with H_2_O_2 _production from other sources. The observed changes in hepatic peroxisomal β-oxidation (Fig. 3) cannot explain the decrease in the catalase activity because peroxisomal β-oxidation did not change in the control mice and even increased in the LPA-transgenic mice on HFHC diet. The increased peroxisomal β-oxidation in LPA transgenic mice on the HFHC diet is in agreement with reports showing that diets high in fat, particular those containing hydrogenated fat, cause increased of peroxisomal β-oxidation [14,15]. In non-transgenic mice the same diet caused no significant increase of peroxisomal β-oxidation. It follows that mice, transgenic with respect to LPA may be more prone than non-transgenic mice to increase of peroxisomal β-oxidation probably because of the low fat content (Fig. 2).

The observed increase in polyamine oxidase and β-oxidase activity together with a decrease in catalase activity indicate that also other oxidative processes are changed as a result of the introduction of the LPA gene. This seems interesting in the view of the possible role of oxidized phospholipids as modulators of inflammatory processes. Thus, modified phospholipids accumulate at sites of inflammation such as atherosclerotic lesions [16]. Furthermore, oxidized phospholipids are shown to induce expression of atherosclerosis-related genes [17]. Additional evidence has been provided by the use of murine natural monoclonal IgM antibody, EO6, which binds to oxidation-specific epitopes on oxidized low-density lipoprotein. A high correlation between plasma Lp(a) and EO6 was then found [18].

The conclusion so far must be that changes in the three peroxisomal enzyme activities in LPA-transgenic mice is more likely to be caused by altered lipid metabolism in these animals, rather than by a direct effect of the LPA construction on the expression of the peroxisomal genes.

### Polyamines

In the kidneys, neither the LPA gene nor the HFHC diet and AG treatment seemed to influence the polyamine levels significantly (data not shown).

The hepatic spermidine and spermine concentration were almost unaffected by aminoguanidine treatment (Fig. 4 and 5). The same was true for the kidneys. Although the polyamine concentration in the standard diet was much higher than in the HFHC diet, the HFHC diet resulted in higher hepatic spermidine concentration. It seems not likely that differences in the intestinal micro flora caused the observed differences in hepatic polyamine content between the two feeding groups.

Clofibrate treatment, that decreases plasma lipid levels has earlier been found to increase the hepatic content of spermidine and reduce that of spermine [9], which is contrary to the effect of the HFHC diet.

The HFHC fed group had higher levels of hepatic fat, as judged by the amount of fat filled vacuoles (steatosis) than those on the standard diet. However, we found no significant correlation between hepatic steatosis and hepatic levels of spermidine or spermine. In spite of that, we suggest that hepatic lipid level may be of importance for the hepatic polyamine level. The fact that spermine binds to negatively charged lipids, e.g. phospholipids [19] may in part explain the increased spermine concentration in liver.

Spermidine in plasma can bind to HDL [20], which is known to inhibit the formation of oxidized low-density lipoprotein (LDL) [21]. Polyamines, especially spermine, are also potent antioxidants and anti-inflammatory agents [5]. The polyamine concentrations have further been reported to inhibit platelet aggregation in hypercholesterolemic rabbits [6]. Studies of the role of polyamines in atherogenesis/thrombogenesis are therefore called for.

The spermidine/spermine ratio is known to be high in fast growing tissue. Thus, in rat liver at birth the ratio is 4.5, whereas this ratio at 9 months is 0.8 [22]. One can only speculate if the cell turnover rate for liver cells in mice fed the HFHC diet, that have a hepatic spermidine/spermine ratio on 0.76, is reduced compared to that of mice on a standard diet where the corresponding ratio was 1.32.

### Liver steatosis

For two of the feeding groups there was significantly lower degree of hepatic steatosis in the transgenic mice than in the non-transgenic animals (Fig 7) suggesting differences in lipid metabolism. We have earlier reported a significantly higher rate of aortic lesions in LPA transgenic mice than in non-transgenic animals and a positive correlation between apo(a) level and size of aortic lesions [2,3]. This, together with the fact that inflammatory cells only could be seen in the aortic fat infiltrations, not in that of the liver, indicate that there are different mechanisms involved in the deposition of lipids in aortic lesions and in liver cells.

The AG treated mice on the HFHC diet had less steatosis than the untreated animals but the differences were far from significant. This indicates that glycosylation is not participating in the formation of hepatic steatosis in mice.

## Conclusion

An increase in polyamine oxidase activity in hLPA transgenic mice, and an increase in β-oxidation in transgenic mice fed a HFHC diet, together with a decrease in catalase activity may indicate that also other oxidative processes are changed as a result of the introduction of the LPA gene. Such changes would be of great interest because oxidation products are known to be important in the development of arteriosclerosis.

Changes in the polyamine pattern upon increased fat intake are also noteworthy. However, in this study we have not been able to show any connection between polyamines/polyamine-metabolism and the arteriosclerotic lesion seen in the same mice [2,3]. Further studies are warranted.

## Materials and methods

### Reagents

Horseradish peroxidase (EC 1. 11. 1. 7) (Type VI. RZ between 250 and 330 U/mg), N1-acetyl-spermine, 4-aminoantipyrine, palmitoyl-CoA, NAD^+^, FAD, HEPES, hexanediamine, putrescine 2HCl, spermidine 3HCl, spermine 4HCl, and mannitol were purchased from Sigma Chemical Co. (St. Louis, MO., USA). Perhydrol (H_2_O_2_) was obtained from E. Merck (Darmstadt, Germany). All other reagents were of analytical grade. [1,4-^14^C]-Putrescine dihydrochloride (spes.act. 2.11 GBq/mmol) was obtained from Amersham Pharmacia Biotech, Ltd. (Rainham, Essex, UK).

### Animals and feeding

This stock of mice have also been used in a parallel investigation of effects of the LPA gene on the development of aortic lesions [2,3]. Forty-one transgenic mice with cDNA representing the human LPA gene linked to the mouse transferrin promotor were studied. The mice were of a hybrid genetic background of strain C57BL/6 crossed with strain SJL [2,3]. The original transgenic breeder mice were a generous gift from Dr. Richard M. Lawn, Falk Cardiovascular Research Center, Stanford University, CA., USA. Twenty-nine non-transgenic littermates from breeding between transgenic and non-transgenic mice were used as controls. DNA analyses of white blood cells were performed to ascertain the LPA transgenic status of the mice. The mice were of both sexes and between 49 and 67 weeks of age when the study started (see Table 1).

**Table 1:** Diet, treatments, age and sex distribution of LPA transgenic and non-transgenic mice

	**Non-transgenic mice**			**Transgenic mice**		
**Diet and treatment**	**No.**	**Sex**	**Age (days), mean ± SD**	**No.**	**Sex**	**Age (days), mean ± SD**

**Standard**	12	F 6, M 6	481 ± 46	11	F 4, M 7	469 ± 36
**Standard + aminoguanidine**	9	F 5, M 4	501 ± 55	7	F 3, M 4	498 ± 53
**HFHC**	11	F 8, M 3	467 ± 16	17	F 8, M 9	486 ± 42
**HFHC + aminoguanidine**	7	F 5, M 2	498 ± 51	5	F 2, M 3	476 ± 20

F = females

M = males

Half of the animals were fed a standard mouse diet (RMI (E) SQS, Special Diets Services, Witham, Essex, England) containing 2.6% fat (crude oil), where saturated fat accounted for 20%. The other half of the animals, were fed an atherogenic semi-purified diet high in fat and cholesterol (HFHC diet), containing 1.25% cholesterol, 18.4% regular butter and 0.5% sodium cholate (ICN Pharmaceuticals, Inc. 1731 Asse-Relegem, Belgium). Several of the animals on each diet were given 0.1% (w/v) aminoguanidine in the drinking water (Table 1). All the treatments lasted for 100 days.

The amounts of polyamines in the standard diet were: 110 nmol/g putrescine, 240 nmol/g spermidine and 45 nmol/g spermine. The corresponding figures for putrescine and spermidine in the HFHC diet were 1.0 and 3.5 nmol/g, respectively. Spermine could not be detected.

Females and males were housed separately, with 1–5 mice per cage. They had a 12 hrs. light/dark cycle, a constant temperature of 21°C and a relative humidity of 65%. Sentinel mice were used to run a FELASA-style health-monitoring scheme. The mice were clinically healthy throughout the experimental period. The mice were raised in The Laboratory Animal Unit at Norwegian School of Veterinary Science (accredited by the Association for Assessment and Accreditation of Laboratory Animal Care and Use International, Brussels, Belgium), and they were kept according to the regulations of the Norwegian Gene Technology Act of 1994. The study was approved by the Norwegian Animal Research Authority.

### Blood sampling and preparation of tissue samples for enzyme measurements

The mice were anaesthetized by a subcutaneous injection of 0.05 ml/10 g body-weight of a 1:1 mixture of Fenatyl/Midazolum (Hypnorm "Janssen" diluted 10x with water and Dormicum "Roche" 5 mg/ml) prior to blood sampling, which was done by an incision in *vena saphena*. The mice were afterwards killed by neck dislocation, and tissues for analysis were removed as quickly as possible. After removal, the liver was weighed, and transferred into ice-cold mannitol-medium (300 mmol/L mannitol, 25 mmol/L HEPES, 1 mmol/L EGTA, pH 7.2). A 10% (w/v) homogenate was prepared by 2 strokes in a Potter-Elvehjem homogenizer equipped with a Teflon^® ^piston. The homogenate was centrifuged for 1 min at 1075 × g_av_. The resulting supernatants were transferred into several small vials, and stored at -20°C until analyzed.

A 20 cm long proximal segment of the small intestine, starting about 0.5 cm from the pyloric sphincter, was prepared. It was cut open longitudinally, rinsed with ice-cold 0.9% (w/v) NaCl, blotted against filter paper, and homogenized for 15 s in ice-cold mannitol medium with an Ultra-Turrax^® ^at 25,000 rpm. The resulting homogenate was subsequently centrifuged at 1075 × g_av _for 15 min and the supernatant was stored at -20°C until it was analyzed. Prior to assays, one part of the homogenate was mixed with one part of ice-cold mannitol medium, and then Triton X-100 was added to a resulting concentration of 0.05% (v/v).

### Enzyme assays

Catalase (EC 1.11.1.6) activity was assayed by monitoring the decomposition of H_2_O_2 _at 240 nm and 25°C essentially as described by Bergmeyer *et *al [23]. Catalase is labile in dilute solution. Therefore, homogenates were diluted 10 times with 20 mmol/L potassium phosphate buffer, pH 7.4 immediately prior to analysis.

Peroxisomal β-oxidation was assayed as palmitoyl-CoA-dependent NAD^+^-reduction, according to Hovik and Osmundsen [24]. Polyamine oxidase (polyamine: oxygen oxidoreductase, EC 1.5.3.3) was assayed spectrophotometrically as described by Hayashi et al. [25]. Diamine oxidase, EC 1.4.3.6 was determined radiometrically, as described by Okuyama and Kobayashi [26].

Catalase activity, peroxisomal β-oxidation and polyamine oxidase activity were measured in 6 randomly selected mice from each of 7 groups of animals, whereas all 5 mice in the transgenic group on HFHC diet and AG were examined. As seen in Fig. 3 the numbers of mice in some other groups were also less than 6, due to accidental loss of some samples.

### Polyamine assays

Tissue specimens were weighed and thereafter homogenized in 4 volumes of 5% trichloroacetic acid. Hexane diamine was added as an internal standard. The homogenates were kept on ice for 1 hr, followed by centrifugation at 2°C for 10 min at 5000 g_av_. The supernatant was stored at -20°C until analyzed. The polyamines were dansylated [27,28] and separated by HPLC [29] on a Radial PAK-A column (Waters, Milford, Ma., USA).

### Assay of proteins

Protein assay were carried out using the biuret method [30] with Boehringer Precimat (Boehringer Mannheim, Mannheim, Germany) as protein standard.

### Liver steatosis

Liver tissue for morphometric studies were removed and immediately placed in a buffered formaldehyde solution. The examination was made routinely on sections of liver, in paraffin blocks, stained with Oil red O and counterstained with hematoxylin. Hepatic steatosis was identified by light microscopy as empty vacuoles in the cytoplasm of liver cells (Fig. 7). For definitive identification of lipid, frozen sections from separate specimens were stained with Oil Red O. The same pathologist examined the sections blindly, and all measurements were performed four times. The evaluation of visible fat in the liver cells was assessed using a semi quantitative scale from 0 to 3. Zero indicates that no vacuolated liver cells could be detected. In the slightest degree of steatosis (grade 1) tiny vacuoles are found in small parts of the lobule, whereas in the severe case (grade 3) almost every liver cell is affected and most cells have large vacuoles. "Moderate" (grade 2) denotes fatty changes between that of slight and severe steatosis (Fig. 7).

### Statistical analysis

To calculate the significance of differences between non-transgenic and transgenic mice on the same treatment we used an unpaired, two tailed t-test, employing the GraphPad Prism v.2.0 program (GraphPad software Inc., San Diego, USA).

The values given in the text and legends to Figures are means, with SD as indicated.

## List of abbreviations

AGE, advanced glycosoationtion end products; AG, aminoguanidine; HFHC, diet high in fat and cholesterol; LPA, gene encoding apo(a).

## Authors' contributions

SD was responsible for the testing of transgenicity of animals used in this study. BPB and HO were responsible for measurements of β-oxidation, polyamine oxidase and catalase activity. AS did the pathological examinations. HR measured the polyamines. KB and KAE conceived the study and performed its design and coordination. KAE drafted the manuscript. All authors read and approved the final version of the manuscript.
